# A Systematic Review and Meta-Analysis of Surgery for Retroperitoneal Sarcoma With Vascular Resection

**DOI:** 10.7759/cureus.43544

**Published:** 2023-08-15

**Authors:** Artur Rebelo, Onur Bayram, Maurizio Grilli, Jörg Ukkat, Joerg Kleeff, Ulrich Ronellenfitsch

**Affiliations:** 1 Department of Visceral, Vascular and Endocrine Surgery, Martin Luther University Halle-Wittenberg, Halle, DEU; 2 Library of the Medical Faculty Mannheim, Heidelberg University, Mannheim, DEU

**Keywords:** outcomes, vascular, surgery, meta-analysis, sarcoma

## Abstract

This meta-analysis examines the outcomes of patients undergoing surgery with vascular resection for retroperitoneal sarcoma. A systematic literature search based on Preferred Reporting Items for Systematic Reviews and Meta-Analyses (PRISMA) guidelines was conducted, identifying five comparative retrospective cohort studies published from 2015 to 2021, with a total of 1,417 patients (180 in the vascular resection (VR) group and 1,237 in the control (no VR) group). The meta-analysis found that 30-day morbidity rates, as classified by Clavien-Dindo classification > 3, were higher in the VR group compared to the no VR group (46% versus 25%, odds ratio (OR): 1.84, 95% confidence interval (CI): 0.39-8.69, p=0.44). Local recurrence rates during the follow-up period were similar between the two groups (30% versus 30%, OR: 1.46, 95% CI: 0.50-4.25, p=0.49). However, distant recurrence was more frequent in the VR group (32% versus 8.5%, OR: 2.54, 95% CI: 1.05-6.13, p=0.04). In conclusion, although oncovascular procedures are feasible for patients with retroperitoneal sarcomas, the long-term outcomes appear to be worse when compared to procedures that do not involve vessel resections.

## Introduction and background

Soft tissue sarcoma represents 1% of solid malignancies with over 50 histological subtypes and a wide variance of tumor locations [1]. Infiltration of major blood vessels has historically been considered a criterion of non-resectability in surgical oncology. Nevertheless, with recent advances in surgical techniques, oncovascular surgery has been increasingly and successfully used in urologic, pancreatic, hepatic, and sarcoma surgery [2-5]. Retroperitoneal compartment resection represents the treatment of choice for retroperitoneal sarcoma [6,7]. This means that organs adjacent to the tumor, often the colon, spleen, kidney, or parts of the pancreas, are preemptively resected. Following that concept, close contact or invasion of major blood vessels such as the iliac vessels, aorta, or inferior vena cava (IVC) would require vascular resection and reconstruction [8-10]. Studies on patients with retroperitoneal sarcoma who underwent surgery with vascular resection and reconstruction showed heterogeneous results. In a study of 32 patients who underwent retroperitoneal sarcoma resection with IVC resection and reconstruction, the median overall survival (OS) was 59 months and the median disease-free survival (DFS) was 18 months in the IVC resection group compared to the median OS of 65 months and the median DFS of 18 months in patients who underwent surgery without vascular resection (p=0.519, p=0.604) [11]. In another retrospective series involving 425 patients with retroperitoneal liposarcoma, 5% of the patients had vascular resection. This was associated with a higher rate of major complications (54% versus 25%, p=0.002) and a lower five-year OS (60% versus 81%, p=0.05) [12]. To summarize the contemporary literature, we conducted a systematic review with meta-analysis that compares surgery with vascular resection to surgery without vascular resection for the treatment of retroperitoneal sarcoma.

## Review

Materials and methods

The Preferred Reporting Items for Systematic Reviews and Meta-Analyses (PRISMA) guidelines were followed [13]. Also, the study was registered in the International Prospective Register of Systematic Reviews (PROSPERO) database (CRD42022343901) [14].

Search Strategy

A PubMed/Medline, Cumulative Index of Nursing and Allied Health Literature (CINAHL), Cochrane Library, ClinicalTrials.gov (clinical trials registry), and Web of Science Core Collection database search was performed on studies published between database inception and January 1, 2022. The search strategies can be assessed in the Appendices. Furthermore, the reference lists of the selected studies were manually searched to find relevant articles. Abstracts and full-text reviews were evaluated to assess inclusion eligibility.

Inclusion and Exclusion Criteria

**Figure 1:**
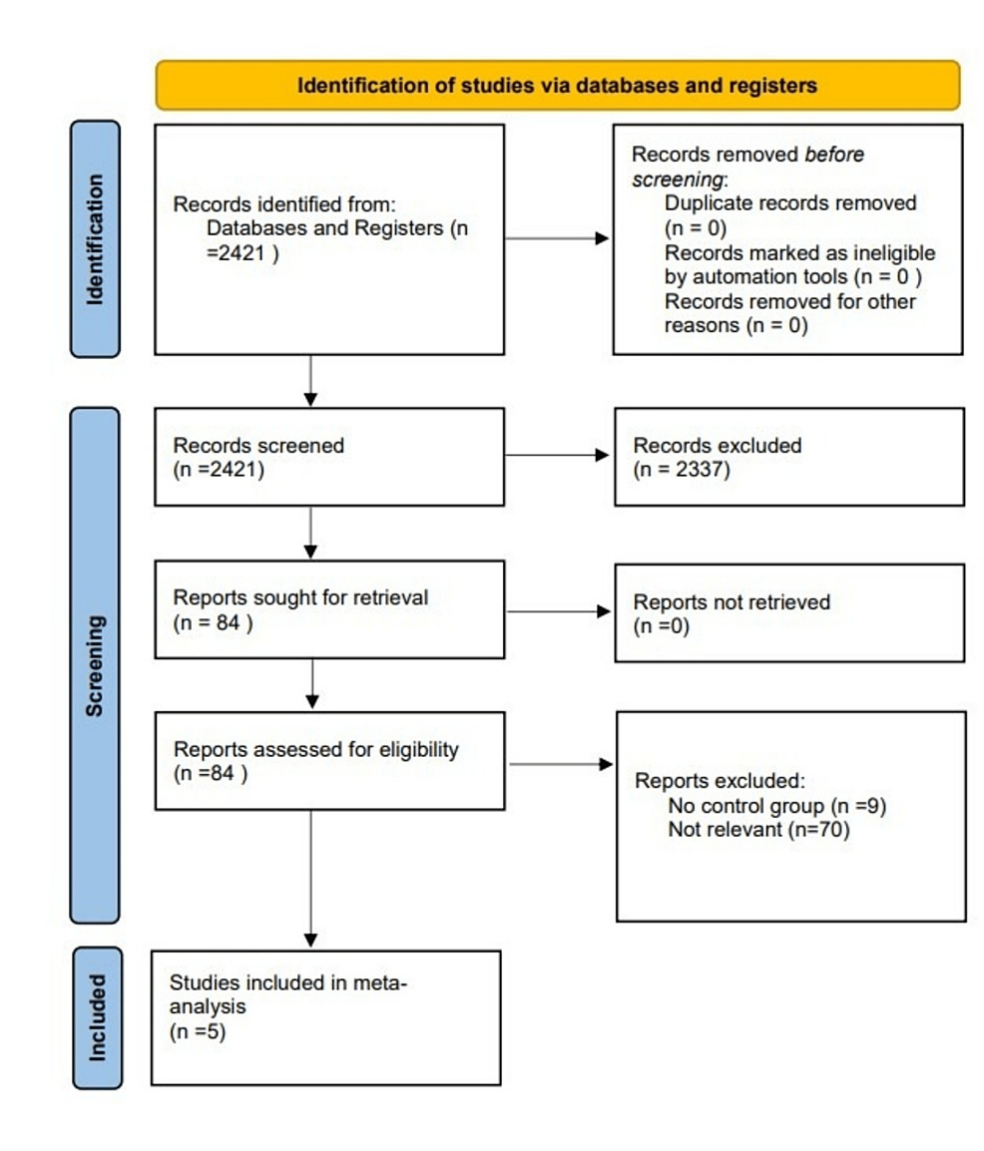
PRISMA flowchart PRISMA: Preferred Reporting Items for Systematic Reviews and Meta-Analyses

Comparative retrospective and prospective studies reporting on the resection of sarcoma, both abdominal and retroperitoneal, including at least one vascular resection group and one group of patients without vascular resection, were included. Studies in the English language were considered. Irrelevant studies, articles reporting on less than five patients, reviews, letters, comments, and case reports were excluded. The study selection process is displayed in a PRISMA flowchart (Figure 1).

Data Collection

Data were extracted by two reviewers (AR and OB) and reverified in case of inconsistencies between the two reviewers. The following data were extracted: first author, year of publication, inclusion period of the study, country and city where the study was conducted, sample size, and mean or median follow-up time in the respective groups. Operation and patient characteristics were also extracted: age, gender, symptoms at presentation, American Society of Anesthesiologists (ASA) classification, comorbidities (diabetes mellitus type 2, chronic obstructive pulmonary disease, chronic renal insufficiency, and coronary heart disease), Eastern Cooperative Oncology Group performance status (ECOG PS), pretreatment biopsy, histological subtype and grade of the sarcoma, preoperative tumor size on CT scan, arterial invasion on CT scan, venous invasion on CT scan, metastatic disease on staging, preoperative radiotherapy, preoperative chemotherapy, type of vessel resection, type of vessel reconstruction, brachytherapy, intraoperative radiotherapy, duration of surgery, blood loss, number of units of red blood cells transfused, length of hospital and ICU stay, pre- and postoperative anticoagulation regime, postoperative radiotherapy and postoperative chemotherapy, patients without any resection upon surgery, and proportion of patients with histologically confirmed vascular tumor invasion. The following outcomes were extracted: mortality (30-day, 90-day, and in-hospital), morbidity (grade ≥3 according to the Clavien-Dindo classification [15]), vascular complications (thrombosis, prosthesis infection, stenosis, or pseudoaneurysm), postoperative bleeding (as defined in the respective study), reoperation rate, median survival time, survival rates (one-, two-, three- and five-year overall, recurrence-free, local recurrence-free, and distant recurrence-free), proportion of macroscopically complete (R0), microscopically incomplete (R1), and macroscopically incomplete (R2) resection, and primary and secondary vessel patency.

Statistical Analysis

If an outcome was reported in at least two studies, a meta-analysis was performed using the Review Manager software version 5.3 (Cochrane Collaboration, Oxford, UK). The magnitude of the effect estimate is presented as forest plots. Weighted mean differences were calculated for continuous data and odds ratios (OR) for binary data. The heterogeneity, statistical significance, and 95% confidence interval (CI) are presented for each outcome. The χ2 and Kruskal-Wallis tests were performed for evaluation of statistical significance (p<0.05). If the studies did not report on mean or standard deviation, the methods described by the guidelines of the Cochrane Collaboration [16] and Hozo et al. [17] were performed. Risk of bias was performed with the Risk Of Bias In Non-randomized Studies of Interventions (ROBINS-I) tool [18].

Results

From the 2,421 articles, five cohort studies from three countries (Italy, France, and the USA) published between 2015 and 2021 were included in the meta-analysis (Figure 1). The enrollment period of these studies ranged from 1994 to 2019. In these studies, 1,417 patients, 180 patients in the vascular resection (VR) group and 1,237 in the control (no VR) group, were included. The median follow-up ranged between 34.4 and 49.2 months. The study features are presented in Table 1.

**Table 1:** Descriptive data from the included studies VR: vascular resection

Study	Group/sample size	Inclusion period	Country/city	Median follow-up (months)
Bertrand et al. (2016) [19]	VR (n=22)	2000-2013	Montpellier/France	34.4
	No VR (n=9)			
Blair et al. (2018) [11]	VR (n=32)	1995-2015	Baltimore, New Haven/USA	37
	No VR (n=96)			
Ikoma et al. (2017) [20]	VR (n=49)	1994-2013	Houston/USA	49.2
	No VR (n=123)			
Tan et al. (2016) [21]	VR (n=67)	1982-2010	New York/USA	39.6
	No VR (n=608)			
Spolverato et al. (2021) [12]	VR (n=24)	2002-2019	Milan/Italy	38
	No VR (n=401)			
Total	VR (n=180)	1994-2019	5 cities/3 countries	Range: 34.4-49.2
	No VR (n=1,237)			

The median age was between 55.5 and 63 years. Across all studies, 71% of patients were female. No information on comorbidities, American Society of Anesthesiologists (ASA) classification, ECOG PS, preoperative CT scan, brachytherapy, intraoperative radiotherapy, length of ICU stay, or anticoagulation regime was provided. Only the study by Spolverato et al. [12] reported symptoms at presentation. Data on pretreatment biopsy and preoperative tumor size was only provided by Bertrand et al. [19]. Regarding the histological type, 895 patients had liposarcoma, 441 patients had leiomyosarcoma, and 156 patients had another histological diagnosis. Only one study reported on preoperative tumor size. All studies reported on possible neoadjuvant therapy. Preoperative radio- or chemotherapy was administered in 12% of patients in the VR group and 14% of patients in the no VR group. The median duration of surgery, median number of units of red blood cells transfused, and median blood loss were also only provided by one study. The IVC was the most frequently resected vessel (67% of all vascular resections) (data from three studies), followed by iliac vessels. Vessel reconstruction was mostly performed with prosthetic grafts (63%) (data from two studies). The median hospital stay was reported as 17 days (VR and no VR groups) by Bertrand et al. [19] and eight days (VR group) by Blair et al. [11]. Adjuvant chemo- and radiotherapy were administered in 7% and 15% of the patients, respectively (data from three studies). Two studies reported the proportion of patients who did not undergo resection: 13% and 6%. Of patients in the vascular resection groups, 78% had histologically confirmed vascular tumor invasion (data from two studies). All included studies reported on the histopathological grade: 27% of tumors were G1 and 73% G2 or G3. The median tumor size was 18.5 cm (data from three studies). The patient and operation characteristics are presented in Table 2 and Table 3.

**Table 2:** Patient and operation characteristics from the included studies VR: vascular resection, no VR: no vascular resection, ASA: American Society of Anesthesiologists, ECOG PS: Eastern Cooperative Oncology Group performance status, CT: computed tomography

Study	Group	Age (median)	Gender (female) (%)	ASA (3 and 4) (%)	ECOG PS	Symptoms at presentation	Comorbidities	Preoperative chemotherapy (%, regimen)	Type of vessel resection	Type of vessel reconstruction	Brachytherapy (%)	Intraoperative radiotherapy (%)	Median duration of surgery (minutes)	Median blood loss (mL)	Pretreatment biopsy (%)	Sarcoma histological type	Preoperative tumor size (median/cm)	Preoperative CT scan	Preoperative radiotherapy (%)
Bertrand et al. (2016) [19]	VR	62.1	45.2	-	-	-	-	16	Iliac/femoral, 67.8%; aorta, 6.5%; superior mesenteric artery/vein, 9.6%; vena cava, 42%; renal veins, 16.1%	No reconstruction, 7%; direct suture, 7%; prosthetic graft, 72.1%; reimplantation, 16.3%	-	-	-	-	100	Leiomyosarcoma, 32.26%; liposarcoma, 54.83%; others, 12.91%	12	-	19
	No VR			-	-	-	-		-	-	-	-	-	-	100			-	
Blair et al. (2018) [11]	VR	63	59	-	-	-	-	19	Vena cava, 100%	No reconstruction, 9%; direct suture, 19%; prosthetic graft, 59%; patch, 13%	-	-	-	2,500	-	Leiomyosarcoma, 81%; liposarcoma, 19%	-	-	22
	No VR	63	54	-	-	-	-	23	-	-	-	-			-	Leiomyosarcoma, 81%; liposarcoma, 19%	-	-	29
Ikoma et al. (2017) [20]	VR	55.5	75	-	-	-	-	22.7	Great vessels/iliac vessel and vena cava, 73%	-	-	-	-	-	-	Leiomyosarcoma, 100%	-	-	11.1
	No VR			-	-	-	-		-	-	-	-	-	-	-		-	-	
Tan et al. (2016) [21]	VR	60	56	-	-	-	-	11	-	-	-	-	-	-	-	Leiomyosarcoma, 23%; liposarcoma, 60%; others, 17%	-	-	4
	No VR			-	-	-	-		-	-	-	-	-	-	-		-	-	
Spolverato et al. (2021) [12]	VR	61	12	-	-	Incidental finding, 8%; mass, 58%; pain, 21%; systemic symptoms, 4%; others, 8%	-	25	Vena cava, 38%; iliac vein, 50%; iliac artery, 29%	-	-	-	480	-	-	Liposarcoma, 100%	-	-	25
	No VR	62	43	-	-	Incidental finding, 14%; mass, 54%; pain, 9%; systemic symptoms, 6%; others, 17%	-	13	-	-	-	-	345	-	-		-	-	19

**Table 3:** Patient and operation characteristics from the included studies VR: vascular resection, no VR: no vascular resection, ASA: American Society of Anesthesiologists, ECOG PS: Eastern Cooperative Oncology Group performance status, ICU: intensive care unit

Study	Group	Median number of units of red blood cells transfused	Median length of hospital stay (days)	Length of ICU stay (median, days)	Anticoagulation regime preoperative (yes/no, regimen)	Anticoagulant regime after discharge (yes/no, regimen)	Postoperative radiotherapy (%, regimen)	Postoperative chemotherapy (%, regimen)	Patients without any resection upon surgery (%)	Histopathological grade	Proportion of patients with histologically confirmed vascular tumor invasion (%)	Median tumor size histology (cm)
Bertrand et al. (2016) [19]	VR	-	17	-	-	-	Yes (R1)	Yes (high grade, large tumors, surgical margins)	13	G1, 6.45%; G2, 41.94%; G3, 41.94%	73	-
	No VR	-			-	-					-	-
Blair et al. (2018) [11]	VR	-	8	-	-	-	-	-	-	G1, 6%; G2, 31%; G3, 63%	-	11.3
	No VR	-	-	-	-	-	-	-	-	G1, 12%; G2, 28%; G3, 60%	-	10.2
Ikoma et al. (2017) [20]	VR	-	-	-	-	-	8.7	15.7	-	G1, 5.2%; G2, 16.9%; G3, 47.7%	-	-
	No VR	-	-	-	-	-					-	-
Tan et al. (2016) [21]	VR	-	-	-	-	-	4	7	6	Low, 36%; high, 64%	-	17
	No VR	-	-	-	-	-					-	
Spolverato et al. (2021) [12]	VR	4	-	-	-	-	0	0	-	G1, 25%; G2 and G3, 75%	83	25
	No VR	0.5	-	-	-	-	0	4	-	G1, 33%; G2 and G3, 67%	-	23

If an outcome was present in more than one study, a meta-analysis was performed. Regarding overall and recurrence-free survival, no meta-analysis could be performed as only one study reported corresponding rates, and the others reported only results of multivariable analyses. Only one of the three studies found that VR was associated with shorter overall and recurrence-free survival. Meta-analysis could also not be performed for mortality (30-day, 90-day, and in-hospital), vascular complications, postoperative bleeding, reoperation rate, one-, two-, three-, and five-year survival rates, R0, R1, and R2 resection rates, and primary and secondary vessel patency. The outcomes of the possible meta-analyses are presented in Table 4, and the risk of bias assessment is presented in Table 5.

**Table 4:** Patient outcomes from the included studies VR: vascular resection, no VR: no vascular resection, HR: hazard ratio, CI: confidence interval

Study	Group	Mortality (30-day) (%)	Mortality (90-day) (%)	Mortality (in-hospital) (%)	Morbidity (≥ 3 according to the Clavien-Dindo classification [13]) (%)	Vascular complications (thrombosis, prothesis infection, stenosis, or pseudoaneurysm)	Postoperative bleeding (as defined in the respective study)	Reoperation rate (%)	Overall distal recurrence rate	Primary vessel patency (%)	Secondary vessel patency (%)	Overall local recurrence rate (%)	One-year survival rate (%)	Two-year survival rate (%)	Three-year survival rate (%)	Five-year survival rate (%)	Overall survival (median months/HR)	Recurrence-free survival (median months/HR)	R0 (%)	R1 (%)	R2 (%)
Bertrand et al. (2016) [19]	VR	-	-	0	36.4	9.2	13.6	13.6	45.5	100	100	9.1	77.4	-	61.3	-	-	18.7	58.1	29	0
	No VR	-	-	0	44	-	0	22	33	-	-	22		-		-	-	-			
Blair et al. (2018) [11]	VR	-	-	-	16	-	-	-	-	92	-	-	75	31	25	-	59	18	44	39	9
	No VR	-	-	-	-	-	-	-	-			-	71	43	25	-	65	18	47	49	12
Ikoma et al. (2017) [20]	VR	-	-	-	-	-	-	-	47	-	-	21	-	-	-	73	99.6; multivariate HR (95% CI), 0.89 (0.33-2.41), p=0.821	50.4; multivariate HR (95% CI), 0.77 (0.37-1.59), p=0.252	65.1	-	6
	No VR	-	-	-	-	-	-	-		-	-		-	-	-					-	
Tan et al. (2016) [21]	VR	-	-	-	-	-	-	-	24 vascular resection (yes/no); multivariate HR (95% CI), 1.2 (0.8-2.0), p=0.43	-	-	39 vascular resection (yes/no); multivariate HR (95% CI), 0.8 (0.5-1.5), p=0.54	-	-	-	-	-	-	50	35	9
	No VR	-	-	-	-	-	-	-		-	-		-	-	-	-	-	-			
Spolverato et al. (2021) [12]	VR	-	-	-	25	-	-	-	20	-	-	45	-	-	-	60	Multivariate HR (95% CI), 5.17 (1.41-18.99), p=0.013	Multivariate HR (95% CI), 6.6 (2.16-20.15), p<0.001	96	-	-
	No VR	-	-	-	54	-	-	-	8	-	-	30	-	-	-	70	-	-	96	-	-

**Table 5:** Risk of bias assessed using the ROBINS-I tool ROBINS-I: Risk Of Bias In Non-randomized Studies of Interventions

Study	Bias due to confounding	Bias in the selection of participants	Bias in the classification of interventions	Bias due to deviations from intended interventions	Bias due to missing data	Bias in the measurement of outcomes	Bias in the selection of the reported result
Bertrand et al. (2016) [19]	Moderate (only vascular resection group defined)	Low (all patients with retroperitoneal sarcoma included)	Low (patients who underwent vascular resection were defined)	Low (intention to treat analysis)	Low (Kaplan-Meier curve presented)	Low (outcomes defined)	Low (outcomes predefined and reported)
Blair et al. (2018) [11]	Low (both groups defined)	Low (included eligible patients defined, baseline characteristics, intervention, and follow-up)	Low (vascular resection was defined)	Low (single intervention of interest (vascular resection))	Low (Kaplan-Meier curve presented)	Low (outcomes defined)	Low (outcomes predefined and reported)
Ikoma et al. (2017) [20]	High (no vascular resection group defined)	High (included vascular resection patients not defined)	High (no clear definition of vascular resections)	High (no clear definition of why vascular resection was performed)	Low (multivariate and survival analysis performed)	Low (multivariate and survival analysis performed)	Low (outcomes predefined and reported)
Tan et al. (2016) [21]	High (no vascular resection group defined)	High (included vascular resection patients not defined)	High (no clear definition of vascular resections)	High (no clear definition of why vascular resection was performed)	Low (multivariate and survival analysis performed)	Low (multivariate and survival analysis performed)	Low (outcomes predefined and reported)
Spolverato et al. (2021) [12]	Low (both groups defined)	Low (included eligible patients defined, baseline characteristics, intervention, and follow-up)	Low (vascular resection was defined)	Low (single intervention of interest (vascular resection))	Low (Kaplan-Meier curve presented)	Low (outcomes defined)	Low (outcomes predefined and reported)

**Figure 2:**
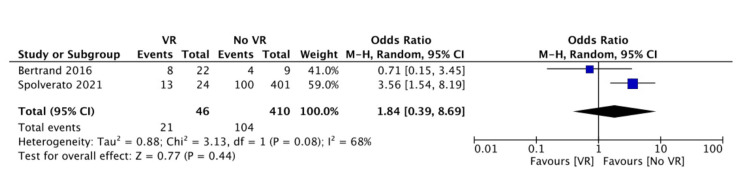
Forest plot of pooled odds ratios with 95% CI for VR versus no VR regarding local recurrence The odds ratios presented are VR versus no VR (with no VR being the reference). VR: patients undergoing surgery for intra-abdominal and retroperitoneal sarcoma with vascular resection, no VR: patients undergoing surgery for intra-abdominal and retroperitoneal sarcoma without vascular resection, CI: confidence interval Bertrand et al. (2016) [19], Spolverato et al. (2021) [12]

**Figure 3:**
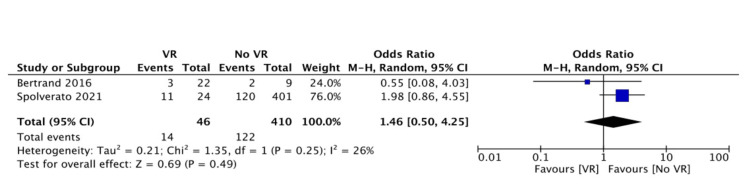
Forest plot of pooled odds ratios with 95% CI for VR versus no VR regarding morbidity The odds ratios presented are VR versus no VR (with no VR being the reference). Morbidity is defined as grade ≥3 according to the Clavien-Dindo classification [13]. VR: patients undergoing surgery for intra-abdominal or retroperitoneal sarcoma with vascular resection, no VR: patients undergoing surgery for intra-abdominal or retroperitoneal sarcoma without vascular resection, CI: confidence interval Bertrand et al. (2016) [19], Spolverato et al. (2021) [12]

**Figure 4:**
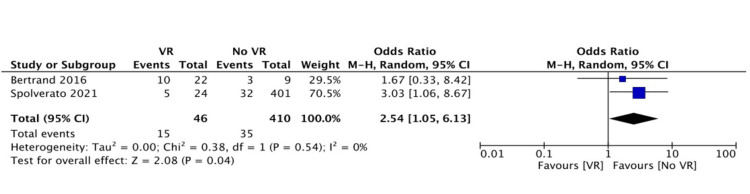
Forest plot of pooled odds ratios with 95% CI for VR versus no VR regarding distant recurrence The odds ratios presented are VR versus no VR (with no VR being the reference). VR: patients undergoing surgery for intra-abdominal and retroperitoneal sarcoma with vascular resection, no VR: patients undergoing surgery for intra-abdominal and retroperitoneal sarcoma without vascular resection, CI: confidence interval Bertrand et al. (2016) [19], Spolverato et al. (2021) [12]

In the meta-analysis regarding 30-day morbidity (Clavien-Dindo classification > 3), higher rates were observed in the VR group (46% versus 25%, OR: 1.84, 95% CI: 0.39-8.69, p=0.44) (Figure 2). Local recurrence rates (during the follow-up of each study) were similar between groups (30% versus 30%, OR: 1.46, 95% CI: 0.50-4.25, p=0.49) (Figure 3). Distant recurrence (during the follow-up of each study) was more frequent in the VR group (32% versus 8.5%, OR: 2.54, 95% CI: 1.05-6.13, p=0.04) (Figure 4).

Discussion

In this systematic review and meta-analysis, we have assessed the impact of vascular resection in surgery for retroperitoneal sarcoma. Despite similar local recurrence rates, higher morbidity and higher distant recurrence rates were observed in the VR group.

Our study is the first to perform a systematic review and meta-analysis on this topic. Due to the rareness of the combination of this pathology and vascular resection, there was a limited number of studies that could be included in our review.

The local recurrence rates from the two studies that we were able to include in the meta-analysis showed no significant differences between vascular resection and no vascular resection [12,19]. Notwithstanding the low statistical power of this analysis, this might show that vascular resection does not increase oncological radicality with regard to the likelihood of local recurrence in the resection of retroperitoneal sarcoma. On the other hand, the high rate of distant recurrence in the VR group must be considered a sign of advanced disease upon resection.

As another main outcome, morbidity rates were significantly higher in the VR group, probably due to the higher complexity of extended oncovascular surgery. Unfortunately, only two studies reported on mortality. Spolverato et al. [12] reported a 90-day mortality of 1% for the whole cohort, and Bertrand et al. [19] reported no mortality in both groups. These results support the notion that radical resection of retroperitoneal sarcoma can be done safely without a high mortality risk.

This meta-analysis has limitations. The main one is that it is exclusively based on retrospective studies with heterogeneous populations and outcome definitions. The PRISMA guidelines were followed [13]. Nevertheless, due to the small number of studies and patients and its retrospective study design, selection bias is a significant limitation of our analysis. The included studies lacked systematic reporting of indications for vascular resection, rendering a correlation with morbidity unfeasible. Also, the results are exclusively based on an uncontrolled non-randomized comparison of patients. Furthermore, since individual patient data were not available, despite our effort in contacting the authors, an estimation of the effects of multimodal therapy on the outcomes was not possible. Therefore, the data should be carefully accessed and used. Furthermore, due to the limited evidence available, no practical clinical recommendations can be made at this time. The strength of our article is that it is the first systematic review and meta-analysis presenting all available studies providing comparative information on the outcome of patients undergoing surgery for retroperitoneal sarcoma with vascular resection with a control group included.

## Conclusions

The current evidence shows that in patients undergoing vascular resection for retroperitoneal sarcoma, despite similar local recurrence rates, higher morbidity and higher distant recurrence rates were observed than in patients who underwent resection of retroperitoneal sarcoma without vascular resection. However, the interpretation of results is limited due to selection bias. Data from prospective controlled studies are needed to be able to better assess the risks and possible benefits of vascular resections in surgery for retroperitoneal sarcoma.

## Appendices

Search strategy and results

PubMed Search (21.01.2022)

Pathology: A systematic search was conducted on PubMed to identify relevant articles related to retroperitoneal neoplasms and sarcoma. The search terms included "Retroperitoneal Neoplasms"[Mesh] OR (("Retroperitoneal Space"[Mesh] OR abdominal*[tw] OR abdomen*[tw] OR Intraabdominal*[tw] OR Retroperitoneal*[tw] OR Retro peritoneal*[tw]) AND ("Sarcoma"[Mesh] OR Sarcom*[tw])). This search yielded a total of 15,855 results.

Intervention: Additionally, articles related to arteries and veins in conjunction with general surgery or surgical procedures were sought. The search terms used were ("Arteries"[Mesh] OR "Veins"[Mesh] OR Arter*[tw] OR Vein*[tw]) AND ("General Surgery"[Mesh] OR "Surgical Procedures, Operative"[Mesh] OR Operat*[tw] OR Surg*[tw] OR Excision*[tw] OR Dissection*[tw] OR resect*[tw] OR removal*[tw] OR ectomy[tw] OR ectomies[tw] OR Preoperat*[tw] OR Postoperat*[tw] OR Perioperat*[tw]), resulting in 631,074 articles.

Cochrane Search (21.01.2022)

Pathology: A search on the Cochrane database focused on retroperitoneal neoplasms and sarcoma using the terms [mh "Retroperitoneal Neoplasms"] OR ([mh "Retroperitoneal Space"] OR abdominal*:ti,ab,kw OR abdomen*:ti,ab,kw OR Intraabdominal*:ti,ab,kw OR Retroperitoneal*:ti,ab,kw OR Retro NEXT peritoneal*:ti,ab,kw) AND ([mh "Sarcoma"] OR Sarcom*:ti,ab,kw), resulting in 171 articles.

Intervention: Arteries and veins in relation to general surgery or surgical procedures were searched using the terms ([mh "Arteries"] OR [mh "Veins"] OR Arter*:ti,ab,kw OR Vein*:ti,ab,kw) AND ([mh "General Surgery"] OR [mh "Surgical Procedures, Operative"] OR Operat*:ti,ab,kw OR Surg*:ti,ab,kw OR Excision*:ti,ab,kw OR Dissection*:ti,ab,kw OR resect*:ti,ab,kw OR removal*:ti,ab,kw OR ectomy:ti,ab,kw OR ectomies:ti,ab,kw OR Preoperat*:ti,ab,kw OR Postoperat*:ti,ab,kw OR Perioperat*:ti,ab,kw), resulting in 51,248 articles.

Web of Science Search (21.01.2022)

Pathology: Articles related to retroperitoneal neoplasms and sarcoma were searched using the terms ("abdominal*" OR "abdomen*" OR Intraabdominal*" OR "Retroperitoneal*" OR "Retro peritoneal*") AND "Sarcom*". This search yielded a total of 3,222 articles.

Intervention: Additionally, articles related to arteries and veins in the context of various surgical procedures were sought using the terms ("Arter*" OR "Vein*") AND ("Operat*" OR "Surg*" OR "Excision*" OR "Dissection*" OR "resect*" OR "removal*" OR "ectomy" OR "ectomies" OR "Preoperat*" OR "Postoperat*" OR "Perioperat*"), resulting in 39,694 articles.

ClinicalTrials.gov Search (21.01.2022)

A search on the ClinicalTrials.gov database aimed to identify relevant clinical trials related to retroperitoneal neoplasms and sarcoma. The search terms included abdominal OR abdomen OR Intraabdominal OR Retroperitoneal OR "Retro peritoneal" AND Sarcom, resulting in 15,422 articles. Additionally, articles related to arteries and veins in conjunction with surgical procedures were sought using the terms (Artery OR Vein) AND (Operation OR Surgery OR Excision* OR Dissection* OR resection OR removal OR ectomy OR ectomies OR Preoperative OR Postoperative OR Perioperative), yielding 11,928 articles.

International Clinical Trials Registry Platform (ICTRP) Search (21.01.2022)

A search on the International Clinical Trials Registry Platform (ICTRP) was performed to identify relevant clinical trials related to retroperitoneal neoplasms and sarcoma. The search terms used were abdominal AND Sarcom OR Intraabdominal AND Sarcom OR Retroperitoneal AND Sarcom OR Retro peritoneal AND Sarcom. However, this search did not yield any results.

## References

[1] Ducimetière F, Lurkin A, Ranchère-Vince D (2011). Incidence of sarcoma histotypes and molecular subtypes in a prospective epidemiological study with central pathology review and molecular testing. PLoS One.

[2] Ghosh J, Bhowmick A, Baguneid M (2011). Oncovascular surgery. Eur J Surg Oncol.

[3] Mulita F, Verras GI, Liolis E (2021). Recurrent retroperitoneal liposarcoma: a case report and literature review. Clin Case Rep.

[4] Verras GI, Mulita F, Bouchagier K (2022). Mid-term outcomes in the treatment of retroperitoneal sarcomas: a 12-year single-institution experience. Med Glas (Zenica).

[5] Tzanis D, Bouhadiba T, Gaignard E, Bonvalot S (2018). Major vascular resections in retroperitoneal sarcoma. J Surg Oncol.

[6] Fairweather M, Gonzalez RJ, Strauss D, Raut CP (2018). Current principles of surgery for retroperitoneal sarcomas. J Surg Oncol.

[7] Bonvalot S, Raut CP, Pollock RE (2012). Technical considerations in surgery for retroperitoneal sarcomas: position paper from E-Surge, a master class in sarcoma surgery, and EORTC-STBSG. Ann Surg Oncol.

[8] Gutlic N, Rogmark P, Nordin P, Petersson U, Montgomery A (2016). Impact of mesh fixation on chronic pain in total extraperitoneal inguinal hernia repair (TEP): a nationwide register-based study. Ann Surg.

[9] Wortmann M, Alldinger I, Böckler D, Ulrich A, Hyhlik-Dürr A (2017). Vascular reconstruction after retroperitoneal and lower extremity sarcoma resection. Eur J Surg Oncol.

[10] Cananzi FC, Ruspi L, Fiore M, Sicoli F, Quagliuolo V, Gronchi A (2021). Major vascular resection in retroperitoneal sarcoma surgery. Surgery.

[11] Blair AB, Reames BN, Singh J (2018). Resection of retroperitoneal sarcoma en-bloc with inferior vena cava: 20 year outcomes of a single institution. J Surg Oncol.

[12] Spolverato G, Chiminazzo V, Lorenzoni G (2021). Oncological outcomes after major vascular resections for primary retroperitoneal liposarcoma. Eur J Surg Oncol.

[13] Moher D, Liberati A, Tetzlaff J, Altman DG (2009). Preferred reporting items for systematic reviews and meta-analyses: the PRISMA statement. PLoS Med.

[14] (2023). Centre for Reviews and Dissemination (CRD): PROSPERO: International Prospective Register of Systematic Reviews. https://www.crd.york.ac.uk/prospero/.

[15] Dindo D, Demartines N, Clavien PA (2004). Classification of surgical complications: a new proposal with evaluation in a cohort of 6336 patients and results of a survey. Ann Surg.

[16] Green S, Higgins J, Alderson P, Clarke M, Mulrow C, Oxman A (2008). Cochrane handbook for systematic reviews of interventions.

[17] Hozo SP, Djulbegovic B, Hozo I (2005). Estimating the mean and variance from the median, range, and the size of a sample. BMC Med Res Methodol.

[18] Sterne JA, Hernán MA, McAleenan A, Reeves BC, Higgins JP (2020). Assessing risk of bias in a non-randomized study. Cochrane handbook for systematic reviews of interventions version 6.1.

[19] Bertrand MM, Carrère S, Delmond L (2016). Oncovascular compartmental resection for retroperitoneal soft tissue sarcoma with vascular involvement. J Vasc Surg.

[20] Ikoma N, Torres KE, Lin HY (2017). Recurrence patterns of retroperitoneal leiomyosarcoma and impact of salvage surgery. J Surg Oncol.

[21] Tan MC, Brennan MF, Kuk D (2016). Histology-based classification predicts pattern of recurrence and improves risk stratification in primary retroperitoneal sarcoma. Ann Surg.

